# Give women what they want: contraceptive discontinuation and method preference in urban Ghana

**DOI:** 10.1186/s40834-022-00200-0

**Published:** 2023-01-16

**Authors:** Sarah D. Compton, Adom Manu, Ernest Maya, Emmanuel S. K. Morhe, Vanessa K. Dalton

**Affiliations:** 1 University of Michigan Department of Obstetrics and Gynecology, 1500 E. Medical Center Dr, Ann Arbor, MI 48109 USA; 2 Department of Population, Family and Reproductive Health, School of Public Health, University of Ghana, P. O. Box LG 13, Legon, Accra, Ghana; 3 University of Health and Allied Sciences Department of Obstetrics and Gynaecology, PMB 31, Ho, Volta Region Ghana

**Keywords:** Contraception, Ghana, Discontinuation

## Abstract

**Background:**

Unmet need for contraception remains high in Ghana. Reducing the number of women who discontinue their contraceptive use is one way to decrease the number of women with an unmet need. In this study, we investigated factors associated with discontinuation among a cohort of Ghanaian women.

**Methods:**

Women who were beginning a new method of contraception at one of six urban clinics in Accra and Kumasi, Ghana were invited to participate in our study. Participants were interviewed before and after their counseling session, and at 3-, 6-, 9-, and 12-months post-enrollment to determine continuation. During follow-up, participants who were no longer using their method were asked why, if they were using any method of contraception, and if so, which method. Logistic regression analysis was performed to identify factors associated with discontinuation for reason other than pregnancy or desired pregnancy.

**Results:**

Of the 472 women who reported leaving their counseling session with a method, 440 (93.2%) had at least one follow-up contact. Of the 440 women, 110 (25%) discontinued their method at some point over the 12-month period, and 94 (85.5%) did so for reasons other than pregnancy or desired pregnancy. In the multivariate regression analysis, women who reported they were given their method of choice were 12.0% less likely to discontinue due to a non-pregnancy reason (*p*=0.005); those who used a long-acting reversible contraceptive (LARC) method were 11.1% less likely (*p*=.001); and those who reported they would choose to use that method again, one measure of satisfaction, were 23.4% less likely (*p*<.001).

**Conclusions:**

To our knowledge, the current study is the first to explore method preference and its relation to continuation. Women in our study who reported they were given the contraceptive method of their choice were less likely to discontinue using that method for non-pregnancy-related reasons. Further, those who adopted a LARC method and those who reported they would make the same method choice again were less likely to discontinue. Women should be supported in selecting a contraceptive method of their choice. Providers should work with their clients to find a method which meets their preferences.

## Background

In Ghana, unmet need for contraception—the discrepancy between a woman’s stated fertility preferences and her actions [1–4]—remains high [5]. This situation persists despite specific efforts by the government, service providers and development partners to enable more women to access family planning services to meet their fertility goals [6]. While unmet need is increasingly seen as a flawed measure [7], and basing the success or failure of family planning programs solely by the numbers of users is problematic, the disconnect between Ghanaian women’s stated fertility goals and their use of contraceptives to meet those goals is one of the largest in sub-Saharan Africa [8]. While increasing new users of contraception is one way to increase use, another focus is reducing the rate of method discontinuation, as many women who start using contraception discontinue their method without switching to another method [9]. Family Planning 2020, a global partnership which aims to reach an additional 120 million women between 2012 and 2020 with contraceptive services, focused on additional users, not “new” or “first time” users—an acknowledgement that decreasing discontinuation and bringing former users back to being current users is also of paramount importance. If women discontinue and do not start a new method while still wanting to avoid pregnancy, they are then at increased risk of an unintended and potentially unwanted pregnancy. In sub-Saharan Africa, Jain and colleagues (2013) estimated that almost one-fifth of women who had ever used contraception discontinued its use, but still had an unmet need for contraception [10].

Method-related discontinuation is not necessarily a negative phenomenon. If a woman starts using a contraceptive method and realizes it does not meet her needs or preferences, she needs to have an avenue to stop using it [9]. If there were an area where discontinuation rates were 0, this would raise concerns about coercion, or an inability for women to stop using methods regardless of whether or not they were satisfied with it. However, women who discontinue a method while still desiring to avoid pregnancy need to be able to switch to another method that is more suitable for them.

In Ghana, the contraceptive prevalence rate among married women is around 27% for all methods and 22% for modern methods [8]. Modern contraceptive use has historically been associated with urban residence [11], having higher levels of education [12] and wealth [13], being married, and being Christian rather than Muslim [14]. Injectables have long dominated the contraceptive method mix in Ghana, and women in two studies reported they are able to be used covertly and that they are convenient, effective, and offer a relatively long interval between administrations [15, 16]. Increasingly, however, implants are gaining popularity in the country, and they may now be the most commonly used method [17]. Overall, there was a steady increase in the contraceptive prevalence rate over the two decades between the late 1980s and the early 2000s, from 7.0% in the 1988 Ghana Demographic and Health Survey (DHS) to 20.8% in the 2003 survey [18]. However, since then, use has plateaued and may be decreasing, especially among highly educated urban women [19]. Ghanaian women report being afraid of side effects at increasingly high rates [20]. Side effects are cited as a reason both for nonuse and for discontinuation of contraception [21–23].

While most Ghanaian women have used a modern method of contraception at some point, discontinuation rates are relatively high [24]. Reasons for contraceptive discontinuation include reliance on short-acting methods [25, 26], desire to become pregnant (which shows self-efficacy in using contraception rather than being indicative of a problem with contraceptive provision), and, increasingly, due to side effects or health concerns. In a study using data from the 2008 Ghana DHS, Modey et al*.* (2014) found that contraceptive method was linked to continuation: implant users were the least likely to have discontinued their method, while IUD users were the most likely [27]. They further found that strong desire to limit births was protective against discontinuation, suggesting the importance of fertility desires and sustained contraceptive use [27].

High levels of method-related discontinuation may indicate low-quality health services; this is an area of study that warrants additional investigation. In this study, we sought to investigate method-related discontinuation among a cohort of Ghanaian women. Specifically, this study seeks to answer: 1) what are the levels of contraceptive discontinuation among a cohort of women in urban Ghana starting a new method of contraception?; 2) what are the reasons for discontinuation?; and 3) what factors, including demographic and satisfaction factors, are predictive of discontinuation?

## Methods

Women who were beginning a new method of contraception at one of six urban clinics in Accra and Kumasi, Ghana during the enrollment period (from September 15 to November 2, 2017) were invited to participate in our study. Eligible participants were referred to study team members by clinic staff, and were approached by trained research assistants for screening and recruitment. Those who were interested in participating were taken to a small room near the waiting area to complete a pre-visit survey. After their counseling session with clinic staff, they were interviewed again, using the post-visit survey. Participants were followed up with phone interviews at 3, 6, 9, and 12 months post-enrollment (December 2017-November 2018) to determine continuation. Records were matched by participant phone number. All interviews were conducted by a team of trained female graduate student research assistants who were assigned to a facility to do initial enrollments and follow-up for that study site. This way, each participant engaged with the same member of the study team for the duration of the study. All study materials and methods were reviewed and approved by the Ghana Health Service Ethical Review Committee and the University of Michigan Institutional Review Board. All participants were taken through a comprehensive oral consent process.

Data were collected electronically on tablets using Qualtrics Mobile, an online survey software. In their pre-visit survey, participants—all women between ages 18 and 49—were asked about their demographics (age, gravidity, parity, marital status, educational level) and questions to assess their previous use of contraception, their knowledge about contraceptive methods, and their method preferences. Participants also completed the Desire to Avoid Pregnancy (DAP) scale [28]. This newly developed scale is designed to capture the sometimes contradictory feelings a woman has about a potential pregnancy. Participants were asked how much they agree with a range of statements such as “If I had a baby in the next year, it would be bad for my life,” “Thinking about having a baby within the next year makes me smile,” and “If I had a baby in the next year, it would be hard for me to manage raising the child.” We included this scale in both the initial and follow-up contacts, as we hypothesized that side effects would be differentially tolerated by women with different levels of desire to avoid pregnancy. For example, women who are highly motivated to avoid pregnancy might be more tolerant of menstrual disruption, while those who hold more positive feelings toward a potential pregnancy might discontinue more readily.

In the post-visit survey, participants were asked if they were leaving with a method, and if so, which method. They were also asked if this was the method they wanted to be using. During follow-up, participants were asked if they were still using the same method they had adopted at the beginning of the study. Those who indicated they were not were asked why, with responses recorded in an open-ended format. During analysis, we coded these responses into method-related factors and pregnancy desire. Examples of method-related factors included “continues bleeding,” “was having a severe headache,” and “have gained weight, prolonged bleeding.” Participants were also asked if they were using any method of contraception, and if so, which method, with responses recorded in an open-ended format. During analysis, these were coded into other modern methods, emergency contraception (EC), condoms, and no method. Participants who were using another modern method were coded as having switched, while those who reported no method or switching to condoms only or EC were coded as having stopped.

Due to the impact of side effects on contraceptive discontinuation rates, all participants were asked at follow-up if they had experienced any side or undesirable effects with their method. Those who answered yes were asked to describe the side effects in an open-ended format. These were coded into menstrual side effects (decreased bleeding, spotting, prolonged bleeding), weight changes, and dizziness/headaches. Participants were further asked if they had been expecting these side effects.

To assess level of satisfaction, at each follow-up encounter, all participants were asked if they received the method they wanted, if they would choose this method again if they had the opportunity, if they would recommend their method to a friend, and their level of decision-making at the initial visit (using the question “During this visit, who made the decisions about what birth control method you would use?” with “The provider,” “Mostly the provider,” “The provider and me together,” “Mostly me,” and “Me” as choices). The response to “would you choose this method again?” was summed across the number of times the participant answered the question and the mean was assessed. Finally, all participants answered the DAP scale at each follow-up, and their mean DAP score across the number of times they answered the DAP was assessed.

### Analysis

Descriptive statistics using frequencies are presented in tables. Bivariate logistic regression analysis was performed to identify factors associated with the outcome variable (discontinued for reason other than pregnancy or desired pregnancy). Factors that were found to be significantly associated at the .1 level, as well as those deemed important to control for, were entered into a multivariate logistic regression analysis. We used marginal effects via the margins command in Stata, as well as odds ratios, to interpret the results of the regression.

## Results

Of 537 women initially enrolled in the study, 472 reported leaving their counseling session with a method; of those, the study team made at least one follow-up contact with 440 (93.2%) of them. These 440 women make up the analytic sample for this analysis. A small majority (243/440, 55.2%) of participants were married, and the vast majority (424/440, 96.4%) had been pregnant at least once. The study team made four follow-up contacts with 233 (53.0%) participants. Other demographic and general information about the sample of women can be found in Table 1.

**Table 1:** Characteristics of sample (*n*=440)

Characteristic	n (%)
Age Group, years	
18-19	20 (4.6)
20-24	122 (28.2)
25-59	123 (28.5)
30-34	83 (19.2)
35+	84 (19.4)
Missing	8 (2.0)
Married	242 (56.4)
Highest Level of Education	
None	62 (14.1)
Primary school	52 (11.9)
Junior secondary school	170 (38.7)
Senior secondary school	102 (23.2)
More than secondary school	53 (12.1)
Missing	1 (.2)
Number of Pregnancies	
None	16 (3.6)
1	92 (21.7)
2	87 (20.5)
3	72 (17.0)
4	62 (14.6)
5	49 (11.6)
6+	62 (14.1)
Number of Follow-Up Contacts	
1	71 (16.1)
2	56 (12.7)
3	80 (18.2)
4	233 (53.0)
Number of Participants at Each Follow-Up	
3 months	404 (75.4)
6 months	321 (59.9)
9 months	321 (59.9)
12 months	309 (57.7)

Of the 440 women, 110 (25%) discontinued their method at some point over the 12-month period. Discontinuations varied by method adopted. While 18.9% (36/190) of those who adopted implants discontinued over the 12 months, 38.5% (5/13) of those who adopted the pill did so. Table 2 shows adoption and non-pregnancy-related discontinuation rates for each method.

**Table 2:** Adoption and discontinuation rates by contraceptive method

Method	Adopted (*n*=440)	Discontinued (*n*=110)
Injection	163 (37.0)	43 (26.4)
Implant	190 (43.2)	36 (18.9)
IUD	38 (8.6)	10 (26.3)
Pill	13 (3.0)	5 (38.5)
Sterilization	3 (0.1)	0 (0)
Missing	33 (7.5)	NA

Data presented as n (%)

Of the 110 women who discontinued their method, 53 (48.2%) did so by 3 months, 22 (20.0%) by 6 months, 25 (22.7%) by 9 months, and 10 (9.1%) by 12 months (Table 3). Of the women who discontinued their method, 94 (85.5%) did so for reasons other than pregnancy or desired pregnancy, with the majority (68, 72.3%) switching to EC, condoms, or no method and only 25 (26.6%) switching to a more effective method.

**Table 3:** Timing, reasons, and action taken by women who discontinued contraception (n=110)

Variable	n (%)
Timing	
By 3 months	53 (48.2)
4-6 months	22 (20.0)
7- 9 months	25 (22.7)
10-12 months	10 (9.1)
Reason	
Pregnancy or desired pregnancy	13 (11.8)
Non-pregnancy-related	94 (85.5)
Missing	3 (2.7)
Action Taken After Discontinuation^a^	
Switched to effective method	25 (26.6)
Switched to EC, condoms, or no method	68 (72.3)
Missing	1 (0.1)

*EC*emergency contraception

^a^Among those who discontinued for non-pregnancy-related reasons (*n*=94)

In the multivariate regression analysis (Table 4), when controlling for marital status, experience of side effects, previous use of contraception, age, and average desire to avoid pregnancy, women who reported they were given the method of contraception they wanted were 12.0% less likely to discontinue due to a non-pregnancy reason; those who used a long-acting reversible contraceptive (LARC) method were 11.1% less likely; and those who reported they would choose to use that method again, one measure of satisfaction, were 23.4% less likely.

**Table 4:** Logistic regression with non-pregnancy-related discontinuation as the outcome variable

Variable	Unadjusted Odds ratio (95% CI)	Adjusted Odds ratio (95%CI)	Marginal effect	Significance
**Got preferred method**	**.479 (.252-.912)**	**.274 (.108-.698)**	**-.120**	**.005**
**LARC**	**.253 (.143-.447)**	**.303 (.143-.642)**	**-.111**	**.001**
**Mean Choose Again**	**.111 (.057-.217)**	**.080 (.031-.206)**	**-.234**	**.000**
Experience side effects	1.99 (1.02-3.84)	1.96 (.926-4.16)	.063	.076
Married	.569 (.349-.929)	.798 (.368-1.73)	-.021	.568
Used a method before this visit	.856 (.522-1.40)	1.34 (.670-2.70)	.027	.405
Average desire to avoid pregnancy	.778 (.526-1.15)	1.06 (.555-1.97)	.004	.891
Age	.958 (.778-1.18)	1.18 (.863-1.62)	.016	.296

Bold indicates statistical significance

*LARC* long-acting reversible contraception

## Discussion

To the best of our knowledge, this is the first prospective study of Ghanaian women to evaluate contraceptive discontinuation. Previous research on this topic has relied almost exclusively on the contraceptive calendar produced by the Ghana DHS Program [29]. Prior evaluations of the calendar method have shown that it underestimates contraceptive use [30], especially for short-term methods [31]. Of the 440 women in urban Ghana who adopted a new method of contraception and were followed up for 12 months, those who reported they were given the contraceptive method of their choice were less likely to discontinue using that method for non-pregnancy-related reasons (p=0.005). Further, those who adopted a LARC method were less likely to discontinue, as were those who reported they would make the same method choice again.

Contrary to findings by Curtis et al. [32], we did not find an association between method-related discontinuation and desire to avoid pregnancy, although this could be because we removed pregnancy-related discontinuations from our model. Similar to Casey et al. in the Democratic Republic of the Congo, we found that the majority of women in our sample were still using their method at 12 months, although our continuation rate of 75 is lower than their 80%. Our finding that women who adopted methods that are user-reliant for continuation—namely, the injectable and the pill—have higher levels of discontinuation than user-independent LARC devices, mirrors findings from other settings [33, 34]. However, this finding is in contrast to an analysis of the 2008 Ghana DHS by Modey et al., where intrauterine device (IUD) users were the most likely to discontinue use. However, implant users in that study were the least likely to discontinue [27]. In our study, over 25% of those who adopted the IUD discontinued over the study period, while 18.9% of implant users discontinued. The proportion of women in our study who discontinued over the 12-month period, 25%, is less than in other studies from Ghana [27] and in 19 countries that found discontinuation rates of 38% in the first year and 55% by the end of the second year [35]. Our 25% rate is also less than was found in urban Senegal, where 34.7% discontinued by the end of 12 months and 53.7% by 24 months [34]. This could be due to our only enrolling women who were seeking services at a hospital-based family planning clinic, rather than being population-level and including women who procure contraception from other locations. It is possible these women are more motivated to avoid pregnancy than women who access services from other locations such as pharmacies. The fact that the women in our study selected LARC devices more than any other method points to them being different than the average Ghanaian contraceptive user.

Our finding that women who report they would use their method again were less likely to discontinue over the study confirms the importance of patient satisfaction and method continuation. Similarly to Cardona and colleagues [29], we found that women who were more satisfied with their contraceptive method were less likely to discontinue use over our study period.

Our finding of only 26.6% of our participants who discontinued their method switching to another highly effective method is higher than the 17% found in urban Senegal [34]. However, even the higher level in our study leads to concerns about unintended pregnancy following discontinuation. Although beyond the scope of the current analysis, the health outcome impacts of contraceptive discontinuation are important to mention. Jain and Winfrey found in their study of 74,313 women that about one-third of all unintended pregnancies occurred in women who had been using contraception, but discontinued use, with only 3-5% of those discontinuations being due to wanting to become pregnant [36]. Ensuring women are given methods that meet their needs and preferences, and are aware of options to switch to a different method if their adopted method does not work for them, is imperative to allow women to avoid unintended pregnancies resulting from contraceptive discontinuation.

The timing of discontinuations, with many (48.2% of all discontinuations) occurring in the first 3 months of use is interesting. Most of those were due to bleeding, which, if patients can endure the menstrual disruptions, often resolve within 3-5 months [37]. This could indicate that more time during counseling needs to be spent discussing potential bleeding changes, and the timing in which those changes can be expected to resolve. Almost none of our participants were seeking contraceptive services prior to a first pregnancy (3.6% had never been pregnant before), which is consistent with a qualitative study conducted in Accra, in which couples were reluctant to use modern methods of contraception until after starting childbearing, out of fear that hormonal methods would negatively impact future fertility [23]. Side effects and health impacts are increasingly being identified as reasons for contraceptive non-use.

While other studies have identified individual-level (e.g., level of education, fertility preferences, age, marital status, and place of residence) and health service-related (e.g., source of method) factors [25, 34], to our knowledge the current study is the first to explore method preference and its relation to continuation.

Unlike some other work [27], we did not find a relationship between women having a history of abortion and discontinuing their contraceptive method.

This study is not without its limitations. While our follow-up rate of 86.4% is lower than we would have liked, it is higher than similar studies; Holt et al. (2019) reached only 59.6% of their sample of Mexican women [38]. The fact that we only have all four contacts for 53% of our sample could bias our discontinuation rate. It is possible that participants who discontinued are more likely to not be included in follow-ups if they did not want to admit to the study team that they were no longer using their method. While all participants were assured during the consent process that participation was not linked to their clinical care, it is possible that women felt they would receive worse care if they admitted to discontinuing their contraception. Improving follow-up rates and increasing the proportion of participants with four contacts are priorities for future work. That we only sampled from family planning clinics in the country’s two largest cities limits our generalizability to other settings, both within and outside Ghana.

## Conclusions

Providing women with their preferred method of contraception is strongly associated with continuation at 12 months. It is clear that providers should engage in counseling which empowers women to choose the method of contraception which best meets their needs. With high and persistent levels unmet need for contraception, ensuring satisfied use and continuation is an important way to improve on women’s ability to reach their fertility goals.

## Data Availability

The datasets used and/or analyzed during the current study are available from the corresponding author on reasonable request.

## Abbreviations

DAP: Desire to Avoid Pregnancy
EC: Emergency contraception
LARC: Long-acting reversible contraception
IUD: Intrauterine device
